# Likelihood of obesity in early and late childhood based on growth trajectory during infancy

**DOI:** 10.1038/s41366-023-01310-8

**Published:** 2023-04-19

**Authors:** George Moschonis, Anela Halilagic, Eva Karaglani, Christina Mavrogianni, Niki Mourouti, Clare E. Collins, Yannis Manios

**Affiliations:** 1 Department of Food, Nutrition and Dietetics, School of Allied Health, Human Services and Sport, La Trobe University, Bundoora, VIC 3086 Australia; 2 Department of Nutrition and Dietetics, School of Health Science and Education, Harokopio University, 17671 Athens, Greece; 3 Department of Nutrition and Dietetics, Hellenic Mediterranean University, Sitia, Greece; 4 School of Health Sciences, College of Health, Medicine and Wellbeing, University of Newcastle, Newcastle, Australia; 5 Institute of Agri-food and Life Sciences, Hellenic Mediterranean University Research Centre, Heraklion, Greece

**Keywords:** Obesity, Risk factors, Nutrition, Paediatrics, Preventive medicine

## Abstract

**Background:**

Childhood obesity rates have reached epidemic levels with Mediterranean countries reporting among the highest numbers globally. Evidence suggests early life factors, including infant growth rate, increase the likelihood of obesity later in childhood. However, optimal rates of infant growth associated with lower odds of future obesity still remain undetermined. The study aim was to determine the optimal infant growth rate associated with a lower likelihood of childhood overweight and obesity.

**Methods:**

Perinatal and anthropometric data collected from 1778 Greek preschool (2–5 years old) and 2294 Greek preadolescent (10–12 years old) children participating in the ToyBox and the Healthy Growth Study (HGS) respectively, was combined for examination. Logistic regression models and receiver operating curves were used to determine the association between infant growth rate and development of childhood overweight and obesity, as well as optimal infant growth rate, respectively.

**Results:**

Rapid weight gain during the first 6 months of life was positively associated with overweight and obesity in preadolescent children (OR:1.36, 95% CI: 1.13–1.63). Optimal cut-off points for several infancy growth rate indices (i.e., WAZ, WLZ, HAZ, BAZ) associated with a lower likelihood of overweight and obesity in preschool years and preadolescence were also identified.

**Conclusions:**

The current findings could possibly set the basis for healthcare professionals and families to better monitor, assess, and control infant growth rates, thus providing another obesity prevention strategy from early life. These findings, however, and the recommended optimal cut-offs need to be confirmed through further prospective research.

## Introduction

The World Health Organization (WHO) defines overweight and obesity as abnormal or excessive fat accumulation that may impair health [1]. Obesity prevalence has tripled since 1975 [1] with the rise of obesity amongst children also reaching epidemic proportions [2]. Childhood obesity is associated with adverse health consequences and an increased risk of comorbidities later into adulthood [3, 4]. Obese children are psychosocially vulnerable, a consequence of being “socially stigmatised”, bullied, teased, and socially isolated [3].

In some European countries, one third of children between the ages of 6 and 9 years are classified as being overweight or obese [5]. The highest prevalence observed is in Mediterranean countries, including Greece, Cyprus, Italy and Spain [5], where approximately 25% of preschool and 45% of preadolescent children were classified as being overweight or obese [6, 7].

Although the pathogenesis of overweight and obesity is complex and multi-factorial [8], the primary aetiology of weight gain is a sustained positive energy balance, whereby energy intake is greater than expenditure [9]. The first 1000 days, in which the foetus and infant are exposed to risk factors that impact on growth, development, and future health status, has been highlighted as a critical period for the development of obesity [10].

Growth rate during infancy is determined by measuring changes in body weight and/or recumbent length between birth and 24 months of age. Common time periods used to assess growth rate are from birth to 6, 12, and 24 months. These measurements allow health professionals and researchers to assess growth from birth until the end of adolescence through the use of appropriate sex-specific growth reference values, including percentiles and standard deviation scores (i.e., z-scores). The change in these percentiles and z-scores over time are used to assess the trajectory of growth and its potential deviation from normal. This may present as either poor or rapid growth and requires appropriate intervention to mitigate the likelihood of negative health impacts.

In this context, recent evidence on the relationship between infant growth rate and overweight and obesity in childhood has identified a positive association between rapid growth during infancy and obesity later in childhood [11]. Rapid infant growth is also positively associated with measures of adiposity, such as body mass index (BMI), waist circumference, and fat mass [11]. However, the optimal rate of infant growth during infancy that is linked to a lower likelihood of developing overweight or obesity later in childhood is still unknown. For this reason, this study investigated the association between infant growth during the first six months of life and the likelihood for subsequent obesity development. The primary aim, however, was to identify the optimal growth rate from birth to six months of age associated with a lower likelihood of obesity development in preschool years and preadolescence.

## Methods

### Study design and populations

The current paper represents a secondary analysis of data from two studies conducted with preschool and preadolescent children in Greece, namely the ToyBox (www.toybox-study.eu) and the Healthy Growth Study (HGS), respectively. ToyBox was a randomised controlled intervention conducted with preschool children (2–5 years) from six European countries. For the current secondary analysis, the ToyBox study data from children living in Greece was utilised. HGS was a cross-sectional epidemiological study conducted with preadolescent children (10–12 years) attending fifth or sixth grade in municipalities within the Greek counties of Attica, Aitoloakarnania, Thessaloniki, and Iraklion. For this secondary data analysis, cross-sectional data collected at baseline from all children was utilised, as well as perinatal data collected retrospectively from birth certificates, health records, and parental recalls.

### Ethics approval

ToyBox study and HGS ethics approval was obtained from the Bioethics Committee of Harokopio University in Athens, Greece, and the Greek Ministry of Education. The ToyBox study was also registered with the clinical trials registry (clinicaltrials.gov, ID: NCT02116296). For the secondary data analyses performed for this work, ethics approval was also granted by the Human Ethics Committee of La Trobe University in Melbourne, Australia (Ethics application no: HEC21291).

### Sampling

The sampling procedure for kindergarten selection in ToyBox included random recruitment from three socioeconomic (SES) levels of municipalities. Children within recruited kindergartens were eligible if (i) they were aged between 2 and 5 years at recruitment (i.e., born between January 2007 and December 2008); (ii) their parents/caregivers provided a signed consent form, and (iii) they were not participating in any other research study or health-oriented project during the academic years 2012–2013 and 2013–2014. Signed parental consent forms to participate in the ToyBox study were collected for 1778 preschool children.

The sampling procedure followed in the HGS involved grouping municipalities into three SES categories, which was then used in combination with preadolescent population proportions to randomly select schools for participation. All 77 primary schools invited to participate in the HGS responded positively. Signed parental consent forms were collected for 2655 children (response rate: 64.1%). Complete socioeconomic, demographic, perinatal, and anthropometric data were collected for 2294 out of the enrolled 2655 children (participation rate: 86.4%). Additional information on the sampling procedures can also be found in previously published work relating to ToyBox study and HGS [12, 13].

### Parental sociodemographic and anthropometric data

In both studies, parents or caregivers reported their socio-demographic and anthropometric data via standardised questionnaires. Socio-demographic data included information on age, ethnicity, and education level. Parents reported anthropometric data (weight and height), which was used to calculate BMI, based on Quetelet’s equation (i.e., weight (kg) divided by height squared (m^2^)). Parent BMI was categorised using the WHO BMI cut-offs of underweight/normal weight (BMI < 25 kg/m^2^), overweight (25 kg/m^2^ ≤ BMI < 30 kg/m^2^) and obese (BMI ≥ 30 kg/m^2^). All interviews were conducted by research assistants, who were trained to minimise interviewer’s effect.

### Perinatal factors

In both studies, perinatal data was obtained from children’s birth certificates or health records and from parent reports. Body weight and recumbent length at birth and 6 months were obtained from health records and were used to calculate weight-for-age (WAZ), length-for-age (LAZ), weight-for-length (WLZ) and BMI-for-age (BAZ) z-scores. Type of delivery (i.e. normal delivery or c-section), gestational age, and infant feeding practices up to 6 months of life was also collected from health records. Pre-pregnancy weight status (BMI) of mothers was obtained through maternal reports of height and weight. In addition, children’s birth weight z-score was used to categorise participants into small for gestational age (SGA: WAZ < 10th percentile), appropriate for gestational age (AGA: 10th < WAZ < 90th percentile), or large for gestational age (LGA: WAZ ≥ 90th percentile) at birth, according to WHO growth charts and related z-score cut-offs [14]. The change in WAZ (ΔWAZ) from birth to 6 months was used to categorise children into those having poor weight gain (ΔWAZ < −1 z-score), normal weight gain (−1 z-score ≤ ΔWAZ ≤ + 1z-score), and rapid weight gain (ΔWAZ > + 1 z-score) during infancy.

### Children’s anthropometric data

Children’s body weight was measured to the nearest 100 g in ToyBox, and to the nearest 10 g in the HGS, using digital scales. Height was measured to the nearest 0.1 cm using portable stadiometers. Weight and height were used to calculate BMI, as per Quetelet’s equation. Age- and sex-specific International Obesity Task Force growth charts and relevant cut-offs were used to categorise children as underweight, normal weight, overweight or obese [15, 16].

### Statistical analysis

Both continuous and categorical variables were used for this secondary data analysis. Continuous variables were tested for the normality of their distribution using the Kolmogorov-Smirnov test and were reported as means and standard deviation (sd). Categorical variables were reported as frequencies (n) and percentages (%). Bivariate logistic regression was used to examine associations between growth rate during infancy with overweight and/or obesity in childhood. Receiver operating curves (ROC) were also used for identifying the optimal cut-off points for the changes in WAZ, LAZ, WLZ and BAZ during the first 6 months of life and their values at 6 months of age, above which the likelihood of overweight and obesity in preschool and preadolescence years is reported with the highest possible sensitivity and specificity. The level of statistical significance was set at *P* < 0.05 with all reported p-values being two-tailed. All statistical analyses were conducted using the SPSS statistical software for iOS (version 25.0).

## Results

The sociodemographic characteristics of youth and their families examined in ToyBox and HGS, respectively, are presented in Table 1. The ToyBox study examined a total sample of 1778 preschool children (4.9 ± 0.3 years old), while the HGS sample comprised 2294 preadolescent children (11.1 ± 0.7 years old). The samples were almost equally split into boys and girls. Almost two thirds of fathers (66.7%) and mothers (62.6%) of ToyBox participants were younger than 42 and 38 years of age, respectively. The opposite was observed for fathers and mothers of HGS participants, the majority of whom were older than 42 (62%) and 38 (60.8%) years of age, respectively. In both studies, more than 85% of children were Greek nationals, while parents of ToyBox participants had a higher educational level of more than 14 years (42.2% fathers and 49.5% mothers), compared to parents of HGS participants (34% fathers and 37.4% mothers). Furthermore, Table 1 also presents data on parental weight status, with 69.9% and 30.1% of fathers and mothers of preschool children in the ToyBox study being overweight or obese. Higher percentages were observed for parents in the HGS, where 74.6% and 39.9% of fathers and mothers of preadolescents were overweight or obese.

**Table 1:** *S*ocio-demographic characteristics of children and their parents examined in ToyBox study and in HGS.

	Preschool children (ToyBox Study) (*N* = 1778)	Preadolescents (Healthy Growth Study) (*N* = 2294)
**Socio-demographics**	Mean (sd)	Mean (sd)
Age (years)	4.9 (0.3)	11.1 (0.7)
	*n* (%)	*n* (%)
Sex		
Girl	868 (48.8)	1153 (50.3)
Boy	910 (51.2)	1141 (49.7)
Father’s Age		
< 42 years	977 (66.7)	872 (38.0)
42–46 years	314 (21.4)	745 (32.5)
> 46 years	174 (11.9)	677 (29.5)
Mother’s Age		
< 38 years	1060 (62.6)	898 (39.2)
38–42 years	456 (27.0)	778 (33.9)
> 42 years	176 (10.4)	618 (26.9)
Ethnicity		
Non-national	105 (5.9)	339 (14.8)
National	1671 (94.1)	1955 (85.2)
Paternal education		
<= 14 years	857 (57.8)	1580 (66.0)
> 14 years	625 (42.2)	813 (34.0)
Maternal education		
<= 14 years	859 (50.5)	1502 (62.6)
> 14 years	841 (49.5)	898 (37.4)
Father’s weight status categories		
Normal weight	429 (30.1)	583 (25.4)
Overweight	719 (50.5)	1248 (54.4)
Obese	276 (19.4)	463 (20.2)
Mother’s weight status categories		
Normal weight	1156 (69.8)	1379 (60.1)
Overweight	346 (20.9)	645 (28.1)
Obese	153 (9.2)	270 (11.8)

Table 2 presents the perinatal characteristics of participants. Pre-pregnancy weight status of mothers was similar in both studies, with 6.5% and 6.8% of mothers in ToyBox and HGS classified as underweight prior to their pregnancy, 15.2% and 14.3% of mothers categorised as overweight, and 4.2% and 4.1% classified as obese, respectively. Almost equal percentages were also observed for the weight gain of mothers during pregnancy according to the IOM (Institute of Medicine) recommendations. In this regard, 29% and 35.3% of mothers in ToyBox and HGS gained weight during their pregnancy that was below IOM’s recommendations, while for 36.2% and 32.3% of mothers in ToyBox and HGS, gestational weight gain was above IOM’s recommendations. Furthermore, more than 80% of children in both studies were born full term (i.e., ≥ 37 weeks of gestation). Regarding size at birth, 7.6% and 12.1% of preschool and preadolescent children in ToyBox and HGS were born SGA, while 7.4% of preadolescents in HGS were born LGA, compared to only 0.9% of preschool children born LGA in ToyBox. In terms of children’s weight gain and breastfeeding practices during the first six months of life, the relevant percentages were almost equivalent in both studies. In this regard, 31.2% of preschool children in ToyBox and 33% of preadolescents in HGS had rapid weight gain from birth until their sixth month of life, while 10.4% and 10.6% of preschool children and preadolescents, respectively, had poor weight gain. In terms of breastfeeding, only 10.1% of preschool children in ToyBox and 8.2% of preadolescents in HGS were exclusively breastfed as infants, until their sixth month of age. Finally, the vast majority of children in both studies (i.e., 72.3% in ToyBox and 66.8% in HGS) had solid food introduced into their diet at 5 to 6 months of age.

**Table 2:** Perinatal factors of children and their parents examined in ToyBox and HGS.

	Preschool children (ToyBox Study) (*N* = 1778)	Preadolescents (Healthy Growth Study) (*N* = 2294)
**Perinatal factors**	*n* (%)	*n* (%)
Mothers’ pre-pregnancy weight status		
Normal weight	1205 (74.0)	1716 (74.8)
Underweight	106 (6.5)	155 (6.8)
Overweight	248 (15.2)	329 (14.3)
Obese	69 (4.2)	94 (4.1)
Gestational weight gain ^**^		
Within IOM	563 (34.8)	744 (32.4)
Below IOM	469 (29.0)	810 (35.3)
Above IOM	586 (36.2)	740 (32.3)
Maternal smoking during pregnancy		
Not smoking	1416 (82.9)	1924 (83.9)
Active smoking	293 (17.1)	370 (16.1)
Gestational age (weeks)		
< 37 weeks	253 (15.5)	438 (19.1)
>= 37 weeks	1383 (84.5)	1856 (80.9)
Birth weight for gestational age		
Appropriate (10th−89th percentile)- AGA	1497 (91.4)	1846 (80.5)
Small (< 10th percentile)- SGA	125 (7.6)	278 (12.1)
Large (> 90th percentile) -LGA	15 (0.9)	170 (7.4)
Weight gain in the first 6 months		
Average (−1 to + 1 z-score difference)	891 (58.3)	1295 (56.5)
Poor (< −1 z-score difference)	159 (10.4)	243 (10.6)
Rapid (> +1 z-score difference)	477 (31.2)	756 (33)
Breast feeding (first 6 months)		
Not exclusive Breast feeding	1553 (89.9)	2107 (91.8)
Exclusive Breast feeding	174 (10.1)	187 (8.2)
Timing of solid food initiation		
<= 4 months	133 (7.5)	393 (17.1)
5–6 months	1286 (72.3)	1522 (66.8)
> 6 months	359 (20.2)	368 (16)

^**^Based on recommendations by the Institute of Medicine (IOM’s 2009 report)^(42)^.

**Fig. 1:**
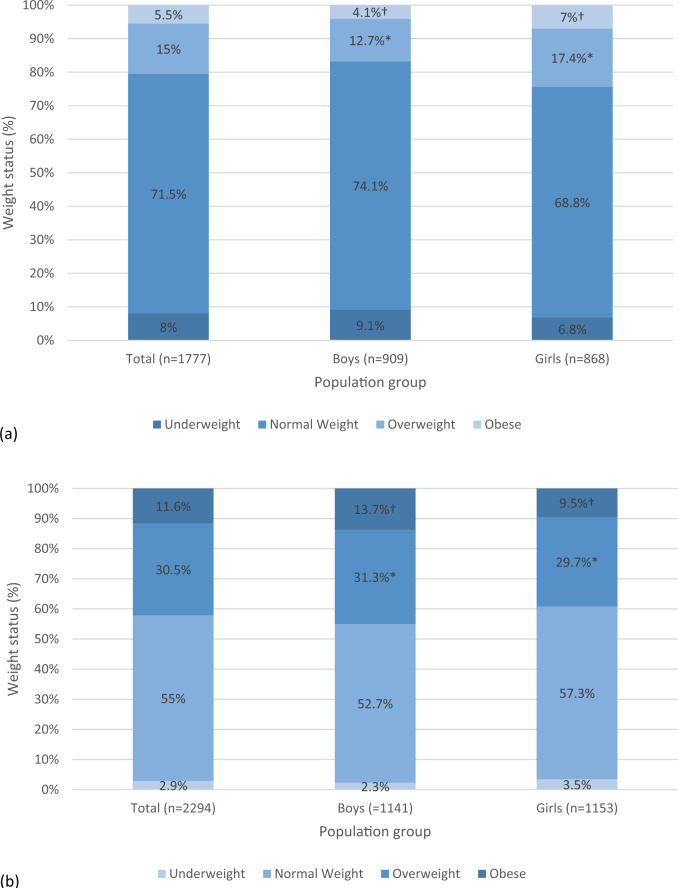
Percentages of children categorised in the different weight status categories, for participants in both studies, as a total sample and by sex. **a** ToyBox study, **b** HGS. *, ^†^Percentages sharing the same superscript symbol are statistically significantly different between boys and girls (*P* < 0.05).

The percentages of children in the different weight status categories, both as a total and by sex, are presented in Fig. 1a for ToyBox and Fig. 1b for HGS. The ToyBox study sample had the higher proportion of children who were of normal weight status, i.e., 71.5%, in comparison to 55% in the HGS. On the contrary, the prevalence rates of overweight and obesity were much higher among preadolescents in the HGS (i.e., 30.5% and 11.6% respectively) compared to preschool children in the ToyBox study (i.e., 15% and 5.5% respectively). When stratifying by sex, the prevalence of overweight and obesity was found to be higher in preschool girls compared to preschool boys in ToyBox (17.4% and 7% for girls vs. 12.7% and 4.1% for boys; *P* < 0.05). The opposite was observed in the HGS, where the prevalence of overweight and obesity was found to be higher in preadolescent boys compared to preadolescent girls (31.3% and 13.7% for boys vs. 29.7% and 9.5% for girls; *P* < 0.05).

The associations between growth rate during the first six months of life and development of overweight or obesity by preschool and preadolescent years are presented as odds ratios (OR) (95% CI) in Table 3. The observed associations were found to be statistically significant only for preadolescents participating in the HGS, showing a higher likelihood of overweight/obesity for infants who had rapid weight gain in their first six months of life (OR: 1.36, 95% CI: 1.14–1.64 for the total population of preadolescents and OR: 1.76, 95% CI: 1.35–2.29 for the subsample of boys in the HGS). A statistically significant association was also observed between poor growth rate and development of overweight/ obesity for preadolescent boys in the HGS (OR: 1.51, 95% CI:1.03–2.20). No statistically significant associations were observed for preschool-aged children in ToyBox.

**Table 3:** Adjusted* odds ratios (95% Confidence Intervals) presenting the associations between overweight/obesity at preschool years and preadolescence and growth rate during the first 6 months of life presented by sex.

ToyBox Study Greek children 2–5 years old	Total (*N* = 1516) OR (95% CI)	Boys (*N* = 778) OR (95% CI)	Girls (*N* = 738) OR (95% CI)
Weight gain in the 6 first months			
Average (−1 to + 1 z-score difference)	Reference	Reference	Reference
Poor (< −1 z-score difference)	0.77 (0.47–1.25)	0.74 (0.38–1.43)	0.80 (0.38–1.69)
Rapid (> +1 z-score difference)	1.20 (0.89–1.62)	1.29 (0.85–1.98)	1.15 (0.75–1.76)

Healthy Growth Study Greek children, 10–12 years old	Total (*N* = 2277) OR (95% CI)	Boys (*N* = 1132) OR (95% CI)	Girls (*N* = 1145) OR (95% CI)
Weight gain in the 6 first months			
Normal (−1 to + 1 z-score difference)	Reference	Reference	Reference
Poor (< − 1 z-score difference)	1.12 (0.85–1.49)	**1.51 (1.02–2.22)**	0.78 (0.51–1.21)
Rapid (> + 1 z-score difference)	**1.36 (1.13–1.63)**	**1.72 (1.31–2.26)**	1.13 (0.88–1.46)

*OR 95% CI: Odds Ratios and 95% Confidence Intervals.

*The analysis was adjusted for size at birth and feeding patterns during infancy.

Bold text indicates statistically significant odds ratios.

The results from the ROC analyses conducted for the identification of the optimal cut-off point values both for the changes of the examined growth indices from birth to six months of age, as well as for the values of these growth indices at six months of age that were associated with the lower likelihood of overweight/obesity with the highest possible sensitivity (Se) and specificity (Sp) in preschool and preadolescent years are presented in Tables 4, 5, respectively. Based on the data presented in Table 4, changes in WAZ, WLZ, and BAZ by 0.54 (Se: 53.8%; Sp: 54.4%), 0.84 (Se: 62.4%; Sp: 51.3%) and 0.82 (Se: 49%; Sp: 59.8%), respectively, were significantly associated with a lower likelihood for the occurrence of overweight or obesity in preschool years. Similar findings were also observed for preadolescents, since changes in WAZ, WLZ, and BAZ by −0.91 (Se: 48%; Sp: 57.8%), 0.89 (Se: 52.9%; Sp: 57.1%) and 0.77 (Se: 53.1%; Sp: 56.6%), respectively, were significantly associated with a lower likelihood for the occurrence of overweight or obesity in preadolescence. No statistically significant findings were observed for the change in HAZ in either study.

**Table 4:** Diagnostic values of growth indices (i.e., WAZ, WHZ, HAZ, and BAZ change) from birth to 6 months of age that are associated with a lower risk of overweight and obesity in preschool and preadolescent years.

	Dependent variable: Overweight/obesity				
	Preschool children 2–5 years old in Greece (ToyBox study)				
Independent variable:	Optimal cut-off point	Sensitivity (%)	Specificity (%)	AUC (95% C.I)	*P*-value
WAZ change	0.540	53.8	54.4	0.546 (0.511–0.581)	**0.012**
WLZ change	0.845	62.4	51.3	0.581 (0.546–0.616)	**<** **0.001**
HAZ change	0.340	48.9	51.7	0.489 (0.453–0.524)	0.536
BAZ change	0.825	49.0	59.8	0.546 (0.510–0.581)	**0.014**

	Preadolescent children 10–12 years old in Greece (Healthy Growth Study)				
Independent variable:	Optimal cut-off point	Sensitivity (%)	Specificity (%)	AUC (95% C.I)	*P*-value
WAZ change	−0.910	48.0	57.8	0.528 (0.503–0.552)	**0.026**
WLZ change	0.890	52.9	57.1	0.551 (0.523–0.529)	**<** **0.001**
HAZ change	−2.685	42.4	57.3	0.489 (0.461–0.516)	0.411
BAZ change	0.770	53.1	56.6	0.553 (0.526–0.580)	**<** **0.001**

*AUC* Area Under the Curve, *WAZ* Weight for Age Z-score, *WLZ* Weight for Length Z-score, *HAZ* Height for Age Z-score, *BAZ* BMI for Age Z-score.

Bold text indicates statistical significance.

**Table 5:** Diagnostic values of growth indices (i.e., WAZ, WHZ, HAZ, and BAZ) at 6 months of age that are associated with a lower risk of overweight and obesity in preschool and preadolescent years.

	Preschool children 2–5 years old in Greece (ToyBox study)				
	Preschool children (ToyBox study, Greek children 2–5 years old)				
Independent variable	Optimal cut-off point	Sensitivity (%)	Specificity (%)	AUC (95% C.I)	*P*-value
WAZ at 6 months	0.585	55.1	71.2	0.668 (0.634–0.702)	**<** **0.001**
WLZ at 6 months	0.105	54.2	68.4	0.635 (0.600–0.671)	**<** **0.001**
HAZ at 6 months	0.930	57.3	52.3	0.571 (0.535–0.607)	**<** **0.001**
BAZ at 6 months	−0.005	53.6	69.0	0.632 (0.596–0.668)	**<** **0.001**

	Preadolescent children 10–12 years old in Greece (Healthy Growth Study)				
Independent variable	Optimal cut-off point	Sensitivity (%)	Specificity (%)	AUC (95% C.I)	*P*-value
WAZ at 6 months	−0.955	46.2	57.7	0.528 (0.503–0.552)	**0.024**
WLZ at 6 months	0.095	50.7	61.1	0.577 (0.550–0.604)	**<** **0.001**
HAZ at 6 months	−2.245	48.8	51.7	0.476 (0.449–0.504)	0.090
BAZ at 6 months	0.230	52.3	59.5	0.575 (0.548–0.603)	**<** **0.001**

*AUC* Area Under the Curve, *WAZ* Weight for Age Z-score, *WLZ* Weight for Length Z-score, *HAZ* Height for Age Z-score, *BAZ* BMI for Age Z-score.

Bold text indicates statistical significance.

According to the results presented in Table 5, values of WAZ, WLZ, HAZ and BAZ at 6 months of age up to 0.58 (Se: 55.1%; Sp: 71.2%), 0.10 (Se: 54.2%; Sp: 68.4%), 0.93 (Se: 57.3%; Sp: 52.3%) and −0.005 (Se: 53.6%; Sp: 69%), respectively, were significantly associated with a lower likelihood for the occurrence of overweight or obesity in preschool years. Furthermore, values of WAZ, WLZ and BAZ at 6 months of age up to −0.95 (Se: 46.2%; Sp: 57.7%), 0.09 (Se: 50.7%; Sp: 61.1%) and 0.23 (Se: 52.3%; Sp: 59.5%), respectively, were significantly associated with a lower likelihood for the occurrence of overweight or obesity in preadolescence.

## Discussion

To our knowledge, this is the first study investigating optimal infancy growth rate associated with a lower likelihood of developing overweight and obesity in later childhood. The association between rapid weight gain and growth during infancy and the occurrence of overweight and/or obesity later in life is well established [11], and broadening the understanding of the optimal growth rate in infancy through further research to mitigate the risk of future obesity will assist with targeted prevention. In this regard, this study examined the association between growth during infancy and the occurrence of overweight or obesity in preschool years and preadolescence. The findings confirmed a strong positive association between rapid growth during the first six months of life and occurrence of overweight or obesity in later years, particularly in preadolescence. Furthermore, optimal cut-offs of growth during infancy that were associated with a lower likelihood for developing overweight or obesity in preschool years and preadolescence were determined.

The positive associations observed in the present study, could be attributed to a combination of early life exposures and other perinatal factors in the HGS study population who demonstrated a higher growth rate in infancy. HGS had a higher prevalence of SGA participants, with fewer being exclusively breastfed compared to the preschool children participating in ToyBox, possibly reflecting a cumulative effect of birth size and feeding practices on infant growth rate. It has been reported that up to 85% of SGA infants experience growth acceleration, otherwise known as “catch-up growth” [17, 18]. Additionally, a study exploring feeding practice-related risk factors for rapid weight gain concluded that breastfeeding during the first six months of life was negatively associated with rapid weight gain, in comparison to the other feeding alternatives [19]. Considering this in the context of this study, children who are born SGA are more likely to be overfed during the first months of their life in order to gain weight, which is associated with an increased risk for the development of overweight or obesity in later years.

Another interesting finding of the present study was also the positive association between poor growth rate during infancy and overweight or obesity in preadolescents. Contrary to catch-up growth that usually occurs in SGA infants, catch-down growth is quite usual in infants born LGA [17]. Catch-down growth in these infants has been reported to have a protective effect from cardiometabolic risk later in life. In this context, Lei et al. [20] found that LGA infants who experienced catch-down growth in the first months of life had lower risk of hypertension at 7 years of age, in comparison to LGA infants without catch-down growth [20]. Other research suggests that stunting or poor linear growth during infancy may also increase the risk of obesity and related comorbidities later in life, as it was found to be associated with lower levels of fat-free mass [21]. Consistent with the literature, the current study indicates that both rapid and poor growth during infancy increases the likelihood of overweight and obesity in childhood and especially in preadolescence, thus highlighting the importance of identifying an optimal rate of growth during infancy as a potential early obesity prevention strategy.

The knowledge gap of what is considered to be the optimal growth rate during infancy has been addressed in the current study. In this regard, optimal cut-offs were reported for several growth indices that can be interpreted as the point at which the probability of developing overweight and obesity during the specified childhood years is reduced. The combination of the two sets of optimal cut-offs allows for the change in an infant’s growth to be tracked from birth to 6 months of age, and then again assessed at 6 months. The combination of the two cut-offs ensures that a recommended limit is placed on the infant’s size at 6 months of age, and that in addition to the change in z-score from birth to 6 months, the z-score at 6 months of age is also used to determine the upper level of growth that is associated with lower odds of overweight and obesity development in later years of life. Although further research is still required, this knowledge may be used to advise clinical practice in paediatrics and paediatric nutrition, as well as population-wide public health initiatives. In this context, the optimal cut-offs could be used in the epidemiological monitoring of infant growth, thus supporting early life prevention initiatives for those infants whose growth does not fall within the optimal growth standards and as such have a higher likelihood to excessively increase their body weight as they grow up.

The findings of the current study should be interpreted considering its strengths and limitations. Regarding strengths, these include the large sample size and the multistage sampling procedure that increased the representativeness of the study population. The examination of two age periods during childhood (i.e., preschool years and preadolescence) could be considered as another strength, allowing the examination of associations between the examined exposures and outcomes at a wider age range. The use of birth certificates and health records to obtain perinatal data, also reduced the potential risk of parental recall bias. Furthermore, the use of trained researchers and standardised procedures to measure and collect anthropometric data in both studies, increased the accuracy of classifying children according to weight status, which otherwise might have been reported inaccurately by parents. Regarding limitations, these mainly include the retrospective collection of perinatal data in both studies, which increase the risk of recall bias. The differences in the two cohorts, in terms of the age groups that were examined (i.e., 2–5 years old in the ToyBox study, 10–12 years old in the HGS), as well as the years when data was collected (i.e., 2012–2014 in the ToyBox study, 2007–2009 in the HGS), could also represent additional limitations of the present study. The retrospectively collected perinatal data in both studies, the moderate sensitivity and specificity values, as well as the modest strength of associations reported, also represent limitations of the current study, since they cannot support a direct causal relationship between growth rate during infancy and obesity in childhood.

In conclusion, the findings of the current study on the association between rapid or poor growth during the first six months of life and the development of overweight and obesity in childhood were in complete alignment with relevant previous research. This further solidifies the understanding of the importance of growth monitoring during the critical time of an infant’s life, especially during the first six months. Additionally, the optimal growth cut-offs identified in the current study could possibly set the basis for healthcare professionals and families to better monitor, assess, and control infant growth rates. However, further research and confirmation of these findings are required before the identified optimal cut-off points can be utilised. If confirmed by other studies, the growth rate cut-offs have the potential to be practically used as part of initiatives for the early detection of high-risk infants and the implementation of obesity prevention initiatives. Consequently, through the implementation of effective early preventive initiatives, obesity-related chronic disease and associated costs on healthcare system will be reduced, while quality of life and lifespan in the population will improve.

## Supplementary information

Dataset 1a, Dataset 1b

## Data Availability

The datasets generated during and/or analysed during the current study are available from the corresponding author on reasonable request.
